# A Versatile Polymer Micelle Drug Delivery System for Encapsulation and *In Vivo* Stabilization of Hydrophobic Anticancer Drugs

**DOI:** 10.1155/2012/951741

**Published:** 2012-02-01

**Authors:** Jonathan Rios-Doria, Adam Carie, Tara Costich, Brian Burke, Habib Skaff, Riccardo Panicucci, Kevin Sill

**Affiliations:** ^1^Intezyne Inc., 3720 Spectrum Boulevard, Suite 104, Tampa, FL 33612, USA; ^2^Novartis Institutes for BioMedical Research, Cambridge, MA 02139, USA

## Abstract

Chemotherapeutic drugs are widely used for the treatment of cancer; however, use of these drugs is often associated with patient toxicity and poor tumor delivery. Micellar drug carriers offer a promising approach for formulating and achieving improved delivery of hydrophobic chemotherapeutic drugs; however, conventional micelles do not have long-term stability in complex biological environments such as plasma. To address this problem, a novel triblock copolymer has been developed to encapsulate several different hydrophobic drugs into stable polymer micelles. These micelles have been engineered to be stable at low concentrations even in complex biological fluids, and to release cargo in response to low pH environments, such as in the tumor microenvironment or in tumor cell endosomes. The particle sizes of drugs encapsulated ranged between 30–80 nm, with no relationship to the hydrophobicity of the drug. Stabilization of the micelles below the critical micelle concentration was demonstrated using a pH-reversible crosslinking mechanism, with proof-of-concept demonstrated in both *in vitro* and *in vivo* models. Described herein is polymer micelle drug delivery system that enables encapsulation and stabilization of a wide variety of chemotherapeutic drugs in a single platform.

## 1. Introduction

It was estimated that there were 1,500,000 new cancer cases and approximately 560,000 deaths from cancer in 2010 [1]. The use of chemotherapy has dramatically improved the survival rate of patients for the last several decades; however, stand-alone chemotherapy drugs suffer from numerous problems including rapid *in vivo *metabolism and/or excretion, inability to access and penetrate cancer cells, and nonspecific uptake by healthy cells and tissue. Often, a large percentage of cytotoxic drug administered to the patient does not reach the tumor environment but rather is distributed throughout the body, resulting in the many toxic effects associated with chemotherapy and a narrowing of the drug's therapeutic window. Polymer micelles offer a promising approach to achieving these goals due to their inherent ability to overcome multiple biological barriers, such as avoidance of the reticuloendothelial system (RES) [2]. Due to their unique size range (20–150 nm), micelles are able to avoid renal clearance (typically less than 20 nm) and uptake by the liver and spleen (particles greater than 150 nm). These micelles can also preferentially accumulate in solid tumors via the enhanced permeation and retention (EPR) effect [3, 4]. The EPR effect is a consequence of the disorganized nature of the tumor vasculature, which results in increased permeability of polymer therapeutics and drug retention at the tumor site.

When considering the design of a nanocarrier, several important factors should be addressed. An ideal delivery system should be composed of biocompatible and biodegradable materials, reproducibly assemble into the desired size range, encapsulate a wide range of drugs and drug classes, maintain particle size in biological media, have the ability to attach cell-specific targeting groups, and release the therapeutic at the site of disease. Polymer micelles have received much attention over the past thirty years as drug delivery vehicle [5–11]. In traditional micelle systems, however, there are no mechanisms in place to keep the micelle intact when it is diluted in the bloodstream, where it is below the critical micelle concentration and interacts with surfactant proteins within the blood. Thus, stability of nanocarriers in biological media remains an issue that needs to be addressed [12]. Some have utilized the approach of chemically conjugating the active drug to a polymer to potentially improve stability. However, this “prodrug” approach is dependent on enzymatic or chemical cleavage of the bond to release the active drug [13–15]. In an attempt to add stability to the micelle, various types of micelles have been developed whereby either the core or shell of the micelle has incorporated crosslinking chemistries, thereby imparting stability at low micelle concentrations [16–22]. However, in many cases, crosslinking is achieved utilizing covalent bonding within the micelle, which does not lend itself to tunable drug release. In addition, in some crosslinked micelles, the crosslinks are physically located with the drug in the core of the micelle, which may interfere with pharmaceutical drug action or drug release from the micelle.

This paper describes a polymer micelle drug delivery system (IVECT) that has effectively addressed the limitations of traditional polymer micelles, by forming micelles that are stable in biological environments. The IVECT triblock copolymer consists of poly(ethylene glycol)-*b*-poly(aspartic acid)-*b*-poly(D-leucine-*co*-tyrosine). The leucine/tyrosine core unit in this polymer is able to encapsulate a wide variety of hydrophobic molecules, which is enhanced by the use of both D and L stereoisomers. The poly(aspartic acid) block was designed to participate in a metal-acetate crosslinking reaction that effectively stabilized drugs inside the core of the micelle and also mediates pH-dependent release of the drug. In this paper, a polymer micelle is described that is composed of biocompatible materials, has the versatility to encapsulate a wide range of therapeutic payloads, is stable to dilution within the blood stream, and has a tunable, highly sensitive, and reversible stabilization mechanism. Data are presented whereby several different hydrophobic molecules are encapsulated and stabilized by crosslinking using a single polymer and without physical manipulation of the drug.

## 2. Materials and Methods

### 2.1. Chemicals and Reagents

All chemicals were obtained from Aldrich or Fisher unless otherwise specified. N_3_-PEG12k-NH-BOC was prepared as described previously [23]. N-carboxy anhydrides (NCAs) were prepared according to previously published procedures. [24, 25]. N-methylpyrrolidone (NMP) was distilled prior to use. BB4007431 and NX-8 were provided by Novartis. Daunorubicin and doxorubicin were obtained from LGM Pharma (Boca Raton, FL). All other drugs were obtained from Yingxuan Pharmaceuticals (Shanghai, China).

### 2.2. Synthesis of Triblock Copolymer

N_3_-PEG12K-NH-Boc (150 g, 12.5 mmol) was dissolved into 1 L of CH_2_Cl_2_/DFA (70/30) and was allowed to stir at room temperature overnight. The product was precipitated twice in diethyl ether and was recovered as a white powder (yield ~ 90%). ^1^H NMR (d_6_-DMSO) 7.77 (3 H), 5.97 (1 H), 3.83–3.21 (1050 H), 2.98 (2 H) ppm.

N_3_-PEG12K-NH_3_/DFA (95 g, 7.92 mmol) was weighed into an oven-dried, 2 L-round-bottom flask and was left under vacuum for three hours before adding the NCA. Asp(OBu) NCA (17.04 g, 79.2 mmol) was added to the flask, and the flask was evacuated under reduced pressure and subsequently backfilled with nitrogen gas. Dry NMP (560 mL) was introduced by cannula, and the solution was heated to 60°C. The reaction mixture was allowed to stir for 24 hours at 60°C under nitrogen gas. Then, D-Leu NCA (24.88 g, 158 mmol) and Tyr (OBzl) NCA (47.08 g, 158 mmol) were dissolved under nitrogen gas into 360 mL of NMP into an oven-dried, round-bottom flask, and the mixture was subsequently added to the polymerization reaction via a syringe. The solution was allowed to stir at 60°C for another three days at which point the reaction was complete (as determined by HPLC). The solution was cooled to room temperature, and diisopropylethylamine (DIPEA) (10 mL), dimethylaminopyridine (DMAP) (100 mg), and acetic anhydride (10 mL) were added. Stirring was continued for 1 hour at room temperature. The polymer was precipitated into diethyl ether (10 L) and isolated by filtration. The solid was redissolved in dichloromethane (500 mL) and precipitated into diethyl ether (10 L). The product was isolated by filtration and dried *in vacuo* to give the block copolymer as an off-white powder (134.6 g, yield = 73%). ^1^H NMR (d_6_-DMSO) *δ* 8.43–7.62 (50 H), 7.35 (100 H), 7.1 (40 H), 6.82 (40 H), 4.96 (40 H), 4.63–3.99 (50 H), 3.74–3.2 (1500 H), 3.06–2.6 (60 H), 1.36 (90 H), 1.27–0.47 (180).

N_3_-PEG12K-*b*-poly(Asp(OBu)_10_)-*b*-poly(Tyr(OBzl)_20_-*co*-D-Leu_20_)-Ac (134.6 g, 6.4 mmol) was dissolved into 1 L of a solution of pentamethylbenzene (PMB, 0.5 M) in trifluoroacetic acid (TFA). The reaction was allowed to stir for five hours at room temperature. The solution was precipitated into a 10-fold excess of diethyl ether, and the solid was recovered by filtration. The polymer was redissolved into 800 mL of dichloromethane and precipitated into diethyl ether. An off-white polymer was obtained after drying the product overnight *in vacuo* (111.8 g, yield = 93%). ^1^H NMR (d_6_-DMSO) *δ* 12.2 (10 H), 9.1 (10 H), 8.51–7.71 (50 H), 6.96 (40 H), 6.59 (40 H), 4.69–3.96 (60 H), 3.81–3.25 (1500 H), 3.06–2.65 (60 H), 1.0–0.43 (180). ^1^H NMR (d_6_-DMSO) *δ* 171.9, 171, 170.5, 170.3, 155.9, 130.6, 129.6, 127.9, 115.3, 114.3, 70.7, 69.8, 54.5, 51.5, 50, 49.8, 49.4, 36.9, 36, 24.3, 23.3, 22.3, 21.2. IR (ATR) 3290, 2882, 1733, 1658, 1342, 1102, 962 cm^−1^. The final composition of the polymer is N_3_-PEG12K-*b*-poly(Asp)_10_-*b*-poly(Tyr_20_-*co*-D-Leu_20_)-Ac, which is also referred to as poly(ethylene glycol)-*b*-poly(aspartic acid)-*b*-poly(D-leucine-*co*-tyrosine).

### 2.3. Micelle Production

All formulations were prepared using oil-in-water emulsion techniques involving dissolving the polymer in water and the drug in an organic solvent. An exemplary formulation technique for daunorubicin follows. The IVECT triblock copolymer (3 g) was dissolved in water (500 mL). Daunorubicin (301 mg) was dissolved in dichloromethane (48 mL) and methanol (12 mL). Just prior to use, triethylamine (0.28 mL) was added to the organic solution to complete the dissolution of the daunorubicin. The aqueous solution was mixed with a Silverson LRT-4 shear mixer (fine emulsor screen, 10,000 RPM). Daunorubicin was added to the mixed solution in a single portion over ~10 s. The solution was mixed for an additional minute and then stirred at room temperature overnight. The resulting solution was then filtered through a 0.22 *μ*m PES filter (Millipore Stericup). Iron (II) chloride solution was added to the concentrated micelle solution at a concentration of 10 mM, and the pH was adjusted to 8.0 and stirred overnight. This solution was frozen on a shell freezer at −40°C and then lyophilized on a Labconco 6 L Plus manifold lyophilization system operating at a pressure of 0.050 Torr and a collector temperature of −85°C. After 48 h, crosslinked, daunorubicin-loaded micelles were recovered as a purple powder (3.22 g, 93% yield).

### 2.4. Drug Weight Loading by HPLC

The mass percentage of active drug within the formulation was determined by HPLC. An exemplary procedure for daunorubicin follows. The daunorubicin-loaded micelle was analyzed by a Waters Alliance separations module (W2695) equipped with Waters Novapak C18, 4 *μ*m column (no. WAT086344) coupled with a Waters Photodiode Array Detector (W2998). Daunorubicin was detected at an absorbance of 480 nm. Mobile phase consisted of a 10 : 70 : 20 ratio of methanol : 10 mM phosphate buffer pH 2.0 : acetonitrile over a 10-minute gradient. Known standards of free daunorubicin were used to determine the percentage by weight of daunorubicin in the formulation (wt/wt%).

### 2.5. Particle Size Analysis

Particle sizes were determined using dynamic light scattering on a Wyatt DynaPro (Santa Barbara, CA). Following lyophilization, micelles were dissolved at 1 mg/mL in 150 mM NaCl and were centrifuged at 2,000 RPM prior to analysis to remove dust.

### 2.6. Encapsulation, Crosslinking, and pH-Dependent Release Dialysis

To test drug encapsulation, the uncrosslinked formulation was dissolved at a concentration of 20 mg/mL in water, which is above the critical micelle concentration of the polymer. Two milliliters were dialyzed in a 3500 MWCO dialysis bag in a volume of 300 mL of 10 mM phosphate buffer, pH 8.0. After dialysis for six hours, the pre- and post-dialysis samples from inside the bag were quantified for drug concentration by HPLC. Encapsulation retention was calculated by dividing the postdrug concentration by the preconcentration.

To test crosslinking, the crosslinked formulation was dissolved in water at a concentration of 0.2 mg/mL, which is below the critical micelle concentration. Three milliliters were dialyzed in a 3500 MWCO dialysis bag in a volume of 300 mL of 10 mM phosphate buffer pH 8. After dialysis for six hours, the pre- and postdialysis samples from inside the bag were quantified for drug concentration by HPLC. Crosslinking retention was calculated by dividing the postdrug concentration by the preconcentration. For pH-dependent release, samples were treated the same as for crosslinking dialysis except for dialysis in 10 mM phosphate buffer pH 3, 4, 5, 6, 7, 7.4, or 8.

### 2.7. *In Vivo* Pharmacokinetic Studies

Female Sprague-Dawley rats weighing about 220 g with jugular vein catheters were obtained from Harlan. Rats were randomly divided into groups of four and were given a single injection of free drug, uncrosslinked drug loaded micelles, or crosslinked, drug loaded micelles dissolved in 150 mM NaCl. Daunorubicin micelles were injected at 10 mg/kg daunorubicin-equivalent dosing, and BB4007431 micelles were injected through the catheter at 25 mg/kg BB4007431 drug-equivalent dosing. Free BB4007431 was dissolved in 0.33 M lactic acid/1.67% dextrose and then diluted in 5% dextrose in water for injection. About 0.25 mL of blood was collected through the catheter at 1, 5, 15 min, 1 h, 4 h, 8 h, and 24 h. Samples were centrifuged at 2000 RPM for 5 minutes to separate plasma. Plasma was then diluted 1 : 4 in cold 0.1% phosphoric acid in methanol with an appropriate internal standard, vortexed for 10 minutes, and centrifuged for 13,000 RPM for 10 minutes. The supernatant was then analyzed by HPLC to determine the drug concentration for each sample. Plasma concentrations were plotted in Microsoft Excel to determine AUC values. Animals were maintained in accordance with *The Public Health Service Policy on Humane Care and Use of Laboratory Animals, *and* the Institutional Animal Care and Use Committee's (IACUC) Principles and Procedures of Animal Care and Use.*

## 3. Results

**Figure 1:**
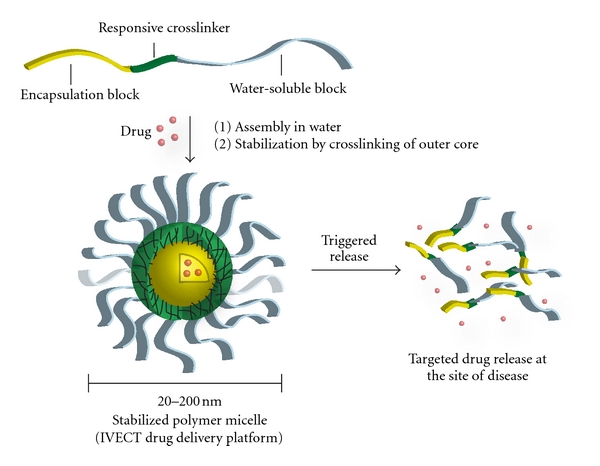
The IVECT polymer micelle. Drugs are loaded into the core hydrophobic block (yellow). The crosslinking block (green) provides stability to the micelle by forming pH-reversible metal-acetate bonds that allow for triggered drug release near the tumor. The PEG block (gray) gives the micelle aqueous solubility and stealth properties *in vivo*.

**Figure 2:**
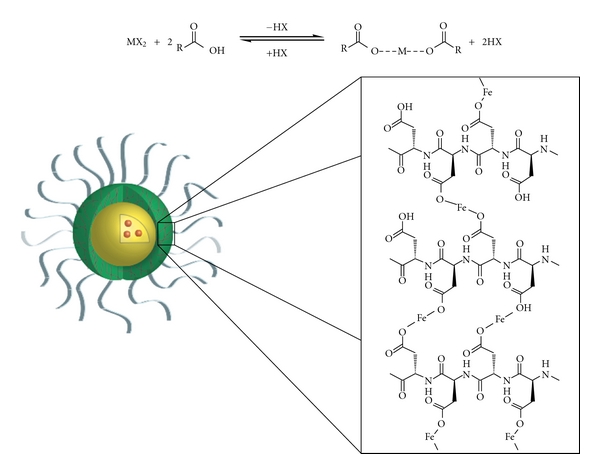
Metal-acetate crosslinking chemistry for stabilization of polymer micelles. While the drug is localized in the core block, the poly(aspartic acid) block of the middle block reacts with metals to form metal acetate bonds. Bonds are formed at high pH and are dissociated at low pH. M represents metal, and X represents a halogen.

The IVECT triblock copolymer consists of poly(ethylene glycol)-*b*-poly(aspartic acid)-*b*-poly(D-leucine-*co*-tyrosine), in which each segment is biodegradable or biocompatible and plays a very important role (Figure 1). Hydrophobic drugs that are loaded into the micelle reside in the encapsulation block (yellow), forming the core of the micelle. The poly(aspartic acid) middle block (green) is the crosslinking block that stabilizes the micelle. In contrast to crosslinking in the core or periphery of the micelle, Intezyne has developed pH-reversible crosslinking technology in the middle block of the triblock copolymer. Crosslinking of this middle layer of the micelle is advantageous since it does not interfere with the core region, which is where the drug resides. The chemistry utilized to crosslink the polymer chains together, and thus stabilizes the micelle, is based on metal acetate chemistry (Figure 2). It is well known that a number of metal ions can interact with carboxylic acids to form metal-acetate bonds [26]. It is also understood that these ligation events form rapidly when the carboxylic acid is in the carboxylate form (e.g., high pH, pH ~ 7-8) yet only weakly interact when the carboxylic acids are fully protonated (e.g., low pH, pH 4-5), therefore allowing release of the drug in low-pH environments, such as regions surrounding the tumor, and the endosomes of tumor cells following endocytosis of micelles. The poly(ethylene glycol) block (Figure 1, shown in gray) allows for water solubility and provides “stealth” properties to the micelle in order to avoid protein opsonization and the reticuloendothelial system [2].

**Figure 3:**
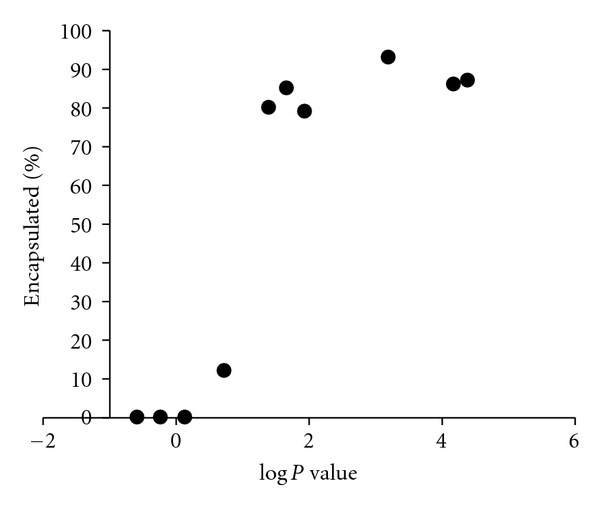
Encapsulation retention of drugs within the micelle is correlated to Log *P* value. The encapsulation retention of the drug, based on an *in vitro* dialysis assay, is plotted compared to its Log *P* value.

As an initial study, the triblock copolymer was used to encapsulate several different small molecule drugs with varying hydrophobicities. A trend was discovered such that the ability of the triblock to encapsulate a drug was dependent on the drug's Log *P* value. Effective encapsulation was achieved with molecules having a Log *P* > 1.4 (Figure 3). The weight loadings of the formulations ranged between 1 and 20%. Molecules that were encapsulated were subsequently crosslinked by the addition of iron chloride. The addition of iron chloride to the micelle did not affect the drug and did not result in generation of polymer-drug conjugates. To test stability of the crosslinked micelle, the *in vitro* stability of the micelle below the CMC was determined using a dialysis assay. In contrast to the encapsulation retention, there was no clear correlation between the Log *P* value and crosslinking retention (Table 1). The particle sizes of crosslinked micelles, as determined by dynamic light scattering, also did not seem related to the Log *P* value. These results demonstrate that the hydrophobicity of the drug influences its ability to be encapsulated within the micelle, but does not influence crosslinking retention or particle size.

**Figure 4:**
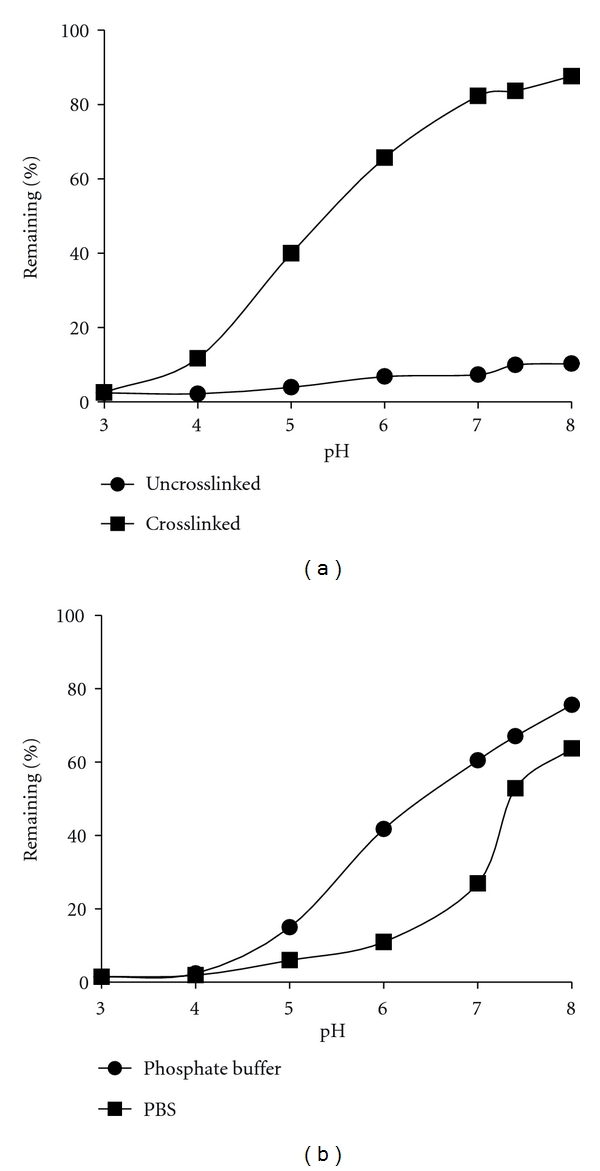
pH-dependent release of drug-loaded micelles. (a) Crosslinked and uncrosslinked BB4007431 micelles were diluted below the CMC and dialyzed for 6 hours in 10 mM phosphate buffer at different pHs. The amount of drug retained before and after dialysis was quantified by HPLC. (b) Crosslinked BB4007431 micelles were diluted below the CMC and dialyzed for 6 hours in either 10 mM phosphate buffer, or PBS, at different pHs. Drug content remaining was quantified by HPLC as above.

To determine whether crosslinked micelles exhibited pH-dependent release, different micelles were dialyzed at concentrations below the CMC in 10 mM phosphate buffer of different pHs. Crosslinked micelles containing BB4007431 demonstrated pH-dependent release of the drug, with increased retention of the drug within the micelle at pH 8, and near total release of the drug after incubation at pH 3 (Figure 4(a)). In contrast, uncrosslinked micelles containing BB4007431 showed nearly complete release of the drug at all pHs, reflecting the instability of the uncrosslinked micelle. To assess the effect of salt in the stability of the micelle, crosslinked BB4007431 was diluted below the CMC and dialyzed in 10 mM phosphate buffer or phosphate-buffered saline (PBS) at different pHs (Figure 4(b)). This experiment showed that salt did destabilize the crosslinked micelle to some degree, but a pH-dependent release was still exhibited.

**Figure 5:**
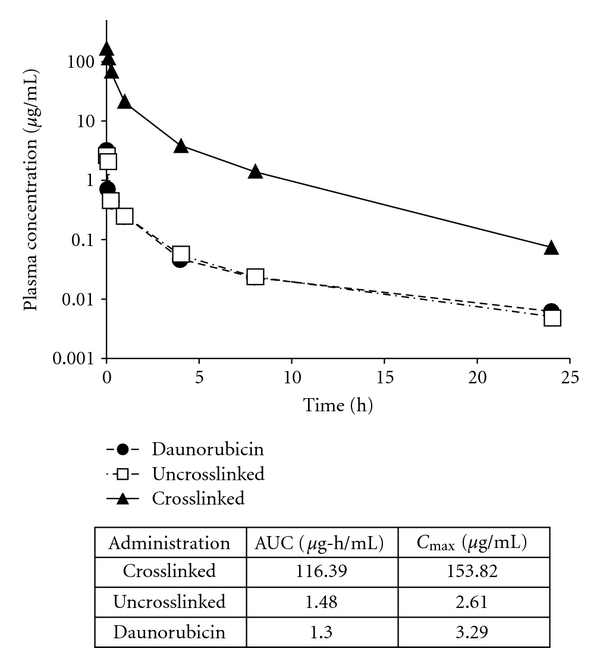
Pharmacokinetics of daunorubicin-loaded micelles in rats. Sprague-Dawley rats were given a single intravenous administration of crosslinked daunorubicin micelle, uncrosslinked daunorubicin micelle, or free daunorubicin at a 10 mg/kg dose. Plasma was analyzed for daunorubicin concentration at various timepoints. The table depicts the area under curve (AUC) and *C*_max_ values for each test article.

**Figure 6:**
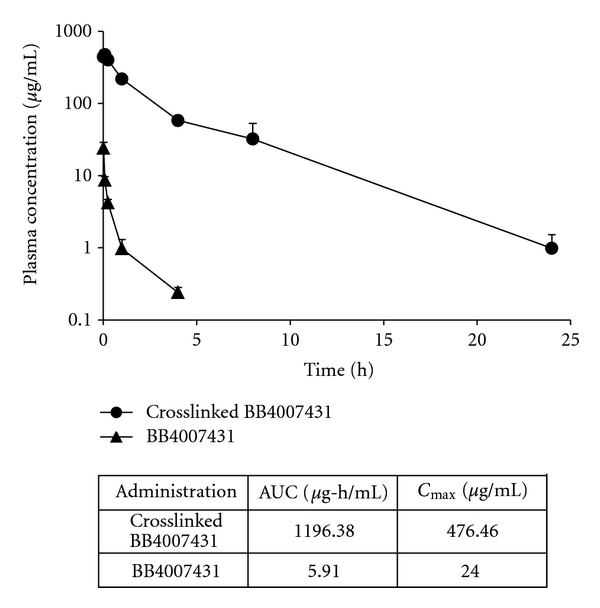
Pharmacokinetics of crosslinked BB4007431 micelles in rats. Sprague-Dawley rats were given a single intravenous administration of crosslinked BB4007431 micelle, or free BB4007431 at a 25 mg/kg dose. Plasma was analyzed for BB4007431 concentration at various timepoints. The table depicts the area under curve (AUC) and *C*_max_ values for each test article.

In order to test the stability of the micelle *in vivo*, a crosslinked, daunorubicin-loaded micelle was assessed in a pharmacokinetic study. Rats were intravenously injected with 10 mg/kg of free daunorubicin, uncrosslinked daunorubicin micelle, or crosslinked daunorubicin micelle, and the concentration of daunorubicin in plasma was determined over the course of twenty four hours (Figure 5). Results demonstrated that the crosslinked daunorubicin micelle exhibited 90-fold increase in plasma in AUC compared to free daunorubicin and 78-fold increase in AUC compared to uncrosslinked daunorubicin. Crosslinked daunorubicin also exhibited a 46-fold higher *C*_max_ than free daunorubicin and a 59-fold increase compared to uncrosslinked micelle. These data demonstrate significantly higher *in vivo* micelle stability with the crosslinked daunorubicin micelle compared to the free drug. A similar study was repeated with a crosslinked formulation of compound BB4007431. Rats injected with crosslinked BB4007431 micelle displayed a vastly superior increase in *C*_max_ (20-fold) and AUC (202.4-fold) compared to free drug (Figure 6). Similar increases in stability were also obtained with crosslinked doxorubicin and paclitaxel-loaded micelles (data not shown), demonstrating the wide applicability of this crosslinking technology to provide increased drug stability *in vivo*.

## 4. Discussion

Improving stability of therapeutic molecules is a well-established aim in the field of drug delivery. An ideal drug-loaded nanoparticle would be stable to dilution in biological media, possess stealth-like properties to avoid uptake by the RES, and release the drug only in the area of diseased tissue. The data presented in this paper describe a versatile polymer micelle drug delivery system that has been engineered to efficiently encapsulate a wide variety of hydrophobic drugs. In addition, the stabilization technology built-in to the micelle is dependent on pH, such that the micelle is stable at physiological pH, and unstable at low pH, thus providing a mechanism to release the drug in the tumor microenvironment or in endosomes, which are both slightly acidic environments.

A vast number of drugs exist today that possess potent anticancer activity; however, many of them are unable to be utilized in the clinic due to their inability to be dissolved in aqueous solutions [27]. Some hydrophobic drugs can be solubilized with excipients; however, such vehicles have been shown to cause toxicity to the patient [28]. The core block of the triblock copolymer (poly(D-leucine-*co*-tyrosine)) was rationally designed and chosen to encapsulate hydrophobic molecules. A key factor leading to the versatility arises from the use of both D and L stereoisomers of amino acids in the core block, which disrupts the secondary structure of the polypeptide. Replacing the rod-like helical nature of the polypeptide with the flexibility of a random coil allows for significant increases in drug loading efficiency. The ability of drugs to be encapsulated within the triblock copolymer was related to its Log *P* value, such that only hydrophobic drugs could be encapsulated. This result is logical as hydrophilic molecules would prefer to associate with the hydrophilic part of the polymer versus the hydrophobic core, leading to inefficient drug encapsulation.

 Crosslinking was performed using metal acetate chemistry, specifically, iron (II) chloride. The crosslinking dialysis assay determined that 40–90% of the drug remained in the crosslinked micelle after six hours. Typically, 10% of the drug or less was retained in uncrosslinked micelles examined using the same crosslinking dialysis assay. Although there was a correlation between Log *P* and encapsulation ability, there was no clear correlation between Log *P* and the crosslinking retention or the particle size. Therefore, it is hypothesized that while hydrophobicity is a strong predictor of success for encapsulation, other variables such as chemical functionality and drug crystallinity play a significant role in micelle size and crosslinking efficiency.

 While stability is important, equally important is the ability to release the drug in a controlled fashion at the site of disease. *In vitro* release assays demonstrated progressive release of drug from the core of the micelle as the pH decreased, which has physiological relevance for delivering drugs to tumors. While passive targeting of nanoparticles within tumor tissue is accomplished by the EPR effect, an additional layer of targeting is possible by employing active targeting strategies, such as decorating the surface of nanoparticles with targeting ligands [29–33]. It is logical to conclude, however, that the ability to target a nanoparticle to tumors is dependent on the stability of the nanoparticle *in vivo*. In pharmacokinetic experiments, superior AUC and *C*_max_ were obtained with several crosslinked micelles, including daunorubicin and BB4007431, compared to their free drug or uncrosslinked micelle counterparts. These data suggest that higher tumor accumulation, and correspondingly improved antitumor efficacy, would be achieved following administration of crosslinked micelle compared to free drug in mouse biodistribution experiments. This would primarily be due to passive targeting by the EPR effect although active targeting has the potential to even further improve delivery of crosslinked micelles.

Polymer micelles hold great promise as drug delivery agents. Indeed, many polymer micelles carrying chemotherapeutic drugs are currently in clinical trials [6, 34]. The utility of a single platform to encapsulate and systemically deliver hydrophobic cancer drugs allows for faster drug screening and facilitated manufacturing processes. In addition to improving the delivery of current anticancer drugs, the polymer micelle system presented herein holds promise for the development of potent, but insoluble novel anticancer drugs. It is envisioned that this new technology will ultimately provide superior treatment options for patients with cancer.

## 5. Conclusions

A polymer micelle drug delivery system was developed that demonstrated encapsulation and stabilization of a wide variety of hydrophobic anticancer drugs. Drug release from stabilized micelles was determined to be pH dependent *in vitro*. *In vivo* pharmacokinetic studies validated increased stability of crosslinked micelles in biological media and demonstrated improved AUC and *C*_max_ compared to uncrosslinked micelles or free drug. These data demonstrate the utility and versatility of a single platform to enable delivery of hydrophobic anticancer drugs to solid tumors.

## Figures and Tables

**Table 1:** Drug formulation properties. The encapsulation retention percentage, crosslinking retention percentage, and particle sizes are shown for eleven compounds tested for loading within the polymer micelle.

Drug	Log *P*	Encapsulation retention (%)	Crosslinking retention (%)	Particle size (nm)
5-Fluorouracil	−0.58	0	NA	ND
Caffeine	−0.24	0	NA	ND
Melphalan	−0.22	0	NA	ND
Gemcitabine	0.14	0	NA	ND
Etoposide	0.73	12	NA	ND
Doxorubicin	1.41	80	63	30
Daunorubicin	1.68	85	78	30
BB4007431	1.94	79	90	55
Paclitaxel	3.2	93	60	36
NX-8	4.18	86	52	86
Vinorelbine	4.39	87	37	47

NA: not applicable, ND: not determined.
